# Uncovering Stability Origins in Layered Ferromagnetic Electrocatalysts Through Homolog Comparison

**DOI:** 10.3390/nano15151210

**Published:** 2025-08-07

**Authors:** Om Prakash Gujela, Sivasakthi Kuppusamy, Yu-Xiang Chen, Chang-Chi Kao, Jian-Jhang Lee, Bhartendu Papnai, Ya-Ping Hsieh, Raman Sankar, Mario Hofmann

**Affiliations:** 1 Graduate Institute of Applied Physics, National Taiwan University, Taipei 10617, Taiwan; opgujela@gmail.com; 2 Department of Electrical and Electronics Engineering, Bakhtiyarpur College of Engineering, Bihar Engineering University, Patna 800001, India; 3 Institute of Physics, Academia Sinica, Taipei 11529, Taiwan; sivasaran413@gmail.com (S.K.); sankarndf@gmail.com (R.S.); 4 Institute of Atomic and Molecular Sciences, Academia Sinica, Taipei 10617, Taiwan; berry45670@gmail.com (Y.-X.C.); jonaslee20@gmail.com (J.-J.L.); yphsieh@gate.sinica.edu.tw (Y.-P.H.); 5 International Graduate Program of Molecular Science and Technology, National Taiwan University, Taipei 10617, Taiwan; 6 Molecular Science and Technology Program, Taiwan International Graduate Program, Academia Sinica, Taipei 10617, Taiwan; 7 Department of Physics, National Taiwan University, Taipei 106319, Taiwan; r13222052@g.ntu.edu.tw; 8 Department of Engineering and System Science, National Tsing Hua University, Hsinchu 300044, Taiwan; bharatpapnai@gmail.com; 9 Nanoscience and Technology Program, Taiwan International Graduate Program, Academia Sinica, Taipei 10617, Taiwan

**Keywords:** electrocatalysis, hydrogen evolution reaction, single-crystal growth, ferromagnetic layered materials, cyclic voltammetry

## Abstract

Magnetic 2D materials offer a compelling platform for next-generation electrocatalysis by enabling spin-dependent reaction pathways. Among them, layered ferromagnets such as Fe_3_GeTe_2_ (FGT) have garnered attention for combining intrinsic ferromagnetism with high predicted oxygen evolution activity. However, the stability of non-oxide ferromagnets in electrochemical environments remains an unresolved challenge, limiting their envisioned applications. In this study, we introduce a structural homolog approach to investigate the origin of FGT’s catalytic behavior and the mechanisms underlying its degradation. By comparing FGT with its isostructural analog Fe_3_GaTe_2_ (FGaT), we demonstrate that the electrochemical activity of FGT arises primarily from Fe orbitals and is largely insensitive to changes in sublayer composition. Although both materials exhibit similar basal-plane hydrogen evolution performance, FGaT demonstrates significantly lower long-term stability. Density functional theory calculations reveal that this instability arises from weaker Te bonding introduced by Ga substitution. These findings establish structural homologs as a powerful strategy for decoupling catalytic activity from electrochemical deterioration and for guiding the rational design of stable magnetic electrocatalysts.

## 1. Introduction

Ferromagnetic electrodes have emerged as a promising class of materials for enhancing electrocatalytic reactions through coupling with a magnetic field. This effect offers potential improvements in efficiency, selectivity, and control of electrochemical reactions [1]. In particular, the magnetic-field-assisted oxygen evolution reaction (OER) has attracted attention for its relevance to energy conversion and conceptual simplicity [2,3,4,5]. Ren et al. demonstrated spin-polarized oxygen adsorption that enhanced the OER kinetics on CoFe_2_O_4_ [6]. Garcés-Pineda linked magnetic improvements of OER over NiZnFe_4_O_x_ to bond alignment of oxygen intermediates [7]. Finally, van der Minne found increased OER activity below the Curie temperature of La_0.67_Sr_0.33_MnO_3_, indicating that bulk magnetic order can couple with surface catalytic sites [8].

These results demonstrate the potential of ferromagnetic electrodes for OER, but they also reveal a common challenge. Suitable electrodes have to fulfill the double requirements of being ferromagnetic and exhibiting sufficient catalytic activity. Previous work has highlighted the potential of two-dimensional (2D) layered magnetic materials for magnetism-assisted electrochemical catalysis [9,10,11]. Two-dimensional, ferromagnetic VSe_2_ has demonstrated promising OER activity, while FePS_3_ is antiferromagnetic and has been applied in HER catalysis [12].

Iron telluride-based compounds, such as Fe_3_GeTe_2_ (FGT), are particularly promising due to the high catalytic activity of FGT surface sites towards OER [13]. Moreover, this class of materials exhibits strong magnetic anisotropy and tunable magnetic properties [14,15,16] that could be exploited in electrochemical catalysis. Isothermal magnetization (M–H) measurements on FGT reveal square-shaped hysteresis loops, high coercivity, and a Curie temperature around 220–230 K, which decreases to ~130 K in monolayer form due to finite-size effects [14,16,17]. In contrast, FGaT exhibits robust ferromagnetism above room temperature, with a Curie temperature near 380 K and magnetization strongly aligned along the c-axis [18]. Off-stoichiometric Fe_3−x_GaTe_2_ displays reduced coercivity and enhanced magnetic entropy change (ΔSₘ ~3.7 J/kg·K), indicating tunable magnetocaloric properties [19].

However, an underappreciated issue of such iron telluride-based ferromagnets is the open question of their environmental stability. Previous work showed the deterioration of FGT in ambient conditions into complex iron oxides [20,21,22] and amorphous layers [17]. Other reports, however, demonstrated the electrochemical stability of transition metal tellurides [23] and demonstrated their suitability for OER [24]. Despite this discrepancy in the literature, prior electrochemical studies of FGT have not addressed its operational stability or proposed strategies to mitigate degradation.

In this work, we establish the origin of the electrochemical properties of FGT by a comparative study involving homologs. Through simultaneous characterization of FGT with its isostructural analog Fe_3_GaTe_2_ (FGaT) we show that the catalytic behavior is not correlated with electrochemical stability. Using combined electrochemical analysis and density functional theory (DFT), we find that Fe orbitals dominate the electrochemical response for both FGT and FGaT, resulting in a comparable electrocatalytic performance. However, the substitution of Ga for Ge was theoretically found to reduce the bonding strength of Te atoms, which agrees with experimental findings on its greater susceptibility to degradation. These insights offer a pathway to decouple activity from stability in magnetic electrocatalysts and provide a route toward their application in future reaction design.

## 2. Materials and Methods

Material weighing, mixing, and sample handling steps were carried out in a nitrogen-filled glove box to prevent oxidation and contamination. Quartz tubes of various dimensions (11 cm × 18 mm, 31 cm × 22 mm, and 41 cm × 22 mm) were used for the synthesis and transport processes. All tubes were evacuated and sealed before heating. Chemical vapor transport was carried out using a two-zone horizontal tube furnace, with specific temperature profiles adjusted for each material: 750 °C/650 °C for FGT and 750 °C/700 °C for FGaT.

XRD measurements were performed using a Bruker D2 PHASER (Billerica, MA, USA) at the NMCGL, Institute of Physics, Academia Sinica. Raman spectra were collected by a NANOBASE XPERRF Raman system (NBOS-220012) by Nanobase Co., Seoul, Republic of Korea with a 532 nm excitation laser using a 0.1 W laser with 3 s exposure. The optical microscopy (Olympus BX53 by Evident Corp. and Olympus Scientific Solutions Americas Corp., Tokyo, Japan). SEM and EDS analyses were conducted on a Hitachi SU8220 at the Instrumentation Center, National Taiwan University.

Electrochemical characterization was carried out using an SP-200 potentiostat/galvanostat (BioLogic Science Instruments, Seyssinet-Pariset, France) and was performed in a standard three-electrode configuration, with Fe_3_GaTe_2_ (FGaT) and Fe_3_GeTe_2_ (FGT) single crystals as the working electrodes, a platinum wire as the counter electrode, and a saturated Ag/AgCl electrode as the reference. All experiments were conducted in 0.5 M H_2_SO_4_ electrolyte under room temperature conditions. Linear Sweep Voltammetry (LSV) was employed to assess the electrocatalytic performance toward the hydrogen evolution reaction (HER). Cyclic Voltammetry (CV) measurements were performed across different scan rates ranging from 10 to 200 mV.

Ab initio simulations were conducted using LCAO-DFT as implemented in Quantum ATK (Synopsys Taiwan Co., Ltd., Hsinchu, Taiwan) provided by the Taiwan Semiconductor Research Institute. Structures were chosen following the previous literature [25], and the unit cell dimension was optimized by using the experimentally obtained lattice constants from XRD. Band structure, total energy, and PDOS calculations were conducted using an unpolarized GGA exchange correlation with a PBE functional. A medium basis set and a 10 × 10 × 3 k-point sampling with a cutoff of 85 Hartree were employed.

## 3. Results

The single-crystal growth of FGT followed a two-step process that began with the synthesis of a polycrystalline precursor. Stoichiometric amounts of Fe, Ge, and Te powders were mixed inside a nitrogen-filled glovebox to prevent oxidation, then sealed in a quartz tube (11 cm length, 18 mm diameter) nested within a larger evacuated tube (31 cm length, 22 mm diameter). The mixture was heated at 650 °C for 24 h, and X-ray diffraction (XRD) confirmed phase purity. This polycrystalline powder was subsequently reground, mixed with 200 mg of iodine as a transport agent, and sealed in a 41 cm × 22 mm quartz ampoule. The ampoule was placed in a dual-zone chemical vapor transport (CVT) furnace with a temperature gradient from 750 °C (hot zone) to 650 °C (cold zone). After 200 h, hexagonal FGT single crystals of several mm size formed in the cold zone [26,27].

**Figure 1:**
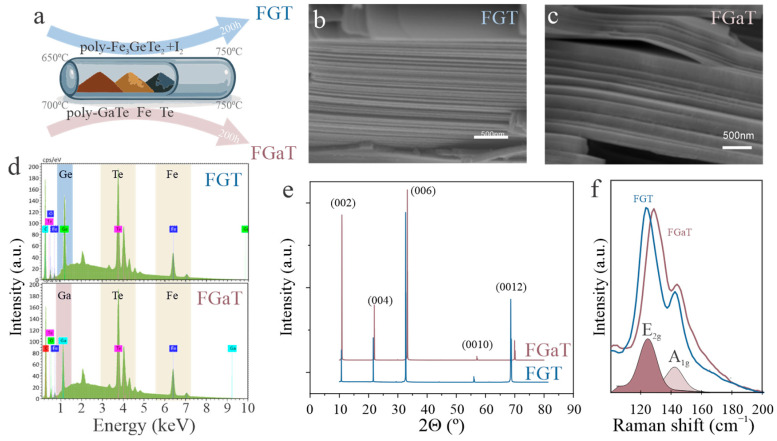
(**a**) Schematic flow chart of the crystal growth processes for FGT and FGaT, (**b**,**c**) representative cross-sectional SEM images of FGT (**b**) and FGaT (**c**), (**d**) EDS spectra of FGT and FGaT, (**e**) XRD patterns of FGT and FGaT, (**f**) Raman spectra of FGT and FGaT with deconvolution of FGT into E_2g_ and A_1g_ modes.

Similarly, the growth of FGaT single crystals involved a multistep synthesis beginning with the formation of GaTe. Stoichiometric Ga and Te powders were mixed under nitrogen, sealed in a quartz tube, and heated at 600 °C for 24 h. Once GaTe formation was confirmed by XRD, it was mixed with Fe and Te powders in stoichiometric proportions to form FGaT. This mixture was sealed in a quartz tube and subjected to a two-step heat treatment at 450 °C and 650 °C for 24 h each, yielding polycrystalline FGaT. For crystal growth, 200 mg of iodine was added as a transport agent, and the powder was sealed in a 41 cm × 22 mm quartz tube under vacuum. The tube was placed in a two-zone furnace with the source region maintained at 750 °C and the growth zone at 700 °C (Figure 1a) (more details can be found in the Supplementary Materials).

To evaluate the microstructure of the grown crystals, scanning electron microscopy (SEM) was employed. Both FGaT and FGT exhibit well-defined layered surfaces with terraced morphologies, characteristic of van der Waals-bonded layered materials (Figure 1b,c). Complementing the SEM analysis, energy-dispersive X-ray spectroscopy (EDS) was used to verify elemental composition. EDS confirmed the stoichiometric presence of Fe, Ge (or Ga), and Te in both types of crystals (Figure 1d) [28,29].

X-ray diffraction analysis provides insights into the crystal structure and orientation. The diffraction pattern of FGT and FGaT matches previously reported results [29,30] and confirms a hexagonal lattice. Both materials exhibit only (00l) reflections, such as (002), (004), and (006), indicating strong c-axis orientation and absence of secondary phases (Figure 1e). The similarity between the FGaT and FGT diffraction patterns suggests that both adopt the same layered hexagonal structure. However, the peaks for FGaT were slightly shifted, corresponding to a roughly 3% increase in the c-axis lattice parameter. This expansion is consistent with the larger ionic radius of Ga relative to Ge.

Raman spectroscopy was performed to further assess the structural similarity of the materials (Figure 1f). For both materials, two major peaks were observed at approximately 125 cm^−1^ and 140 cm^−1^, assigned to the E_2g_ and A_1g_ modes, respectively [25,31]. These correspond to in-plane and out-of-plane vibrations within the layered crystal structure. FGaT exhibits peaks at slightly higher wavenumbers, which suggests stronger bonding and stiffer force constants and validates the effect of elemental substitution on vibrational properties.

With the structural and compositional similarity of our materials verified, we next examine their electrochemical behavior. Recognizing the limitations of conventional methods, we choose a novel approach for electrochemical characterization. Prior studies employed exfoliated or powdered samples dispersed onto conductive substrates for electrocatalytic evaluation [25,32]. However, such configurations fail to differentiate between the intrinsic activity of the basal plane and contributions arising from edges or defect sites. As a result, the observed catalytic performance is frequently dominated by edge effects, which are inherently more reactive due to unsaturated coordination and altered electronic structure [33].

**Figure 2:**
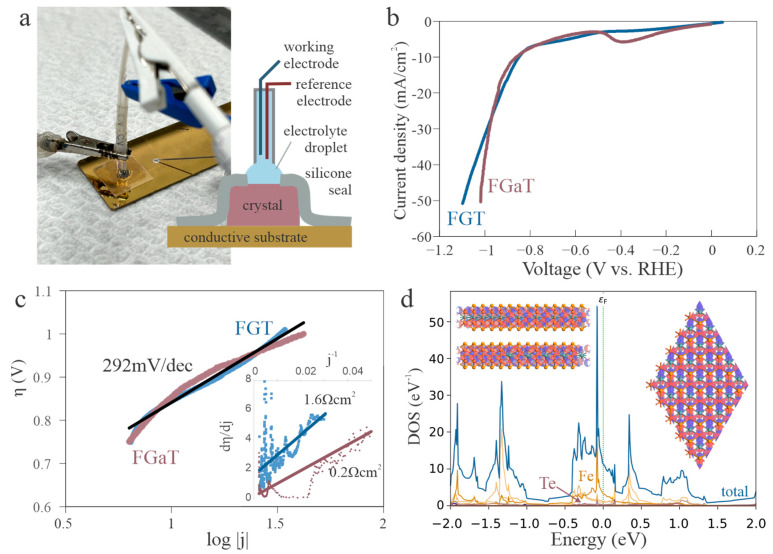
Intrinsic hydrogen evolution performance of basal planes. (**a**) Schematic of the electrochemical device configuration with a silicone membrane used to isolate the basal plane of the single crystal from the edges and substrate; (**b**) Linear sweep voltammetry (LSV) curves in 0.5 M H_2_SO_4_, showing similar overpotentials for FGT and FGaT; (**c**) Tafel analysis confirming that both materials exhibit comparable intrinsic HER kinetics, (inset) graphical characterization of uncompensated resistance according to [34]; (**d**) FGT density of states (DOS) projected onto Fe, Ge, and Te atoms from DFT calculations, (inset) structural model of eigenstate near the Fermi level decomposed into orbital contributions around Fe (red), Te (orange), and Ge (green) atoms confirming dominance of Fe and Te orbitals and minimal contribution of Ge atoms.

To overcome these challenges and isolate the fundamental activity of the basal surface, we exploit the large lateral size of our single crystals. By orienting the crystal to expose only the basal plane and carefully shielding its edges, we minimized edge-related contributions to the electrochemical signal. To implement this design, we fabricated a custom silicone membrane that encapsulates the sample in such a way that only the top basal face remains accessible to the electrolyte, as depicted in Figure 2a. This membrane also prevents direct contact between the electrolyte and the underlying conductive substrate, thereby ensuring that the measured response arises solely from the intrinsic activity of the exposed basal surface of the single crystal.

Using this setup, we conducted linear sweep voltammetry (LSV) in a 0.5 M H_2_SO_4_ solution to examine the catalytic activity of FGT and FGaT. (Raw data and extracted peak parameters are provided in the Supplementary Materials). The measured overpotential for FGT is 0.85 V (Figure 2b), which is significantly higher than values reported in previous studies. This disparity highlights the substantial role that edge sites played in earlier experiments using exfoliates or powders.

Importantly, FGaT exhibits a similar overpotential of 0.88 V, suggesting that the intrinsic basal-plane activity of the two materials is comparable. The slightly steeper slope in the LSV curve for FGaT was found to be due to FGaT’s greater conductivity, which lowers its uncompensated resistance [34] (Inset Figure 2c). Moreover, after resistance correction, the Tafel slopes of both materials converged on the same linear trend, confirming the quantitative similarity of their intrinsic hydrogen evolution kinetics (Figure 2c).

This similarity can be rationalized by considering the underlying catalytic mechanism. Prior studies have shown that the active sites for hydrogen adsorption are associated with top-site geometries on the chalcogen-terminated surface [32]. These sites exhibit limited interaction with the underlying Ga or Ge atoms, suggesting that substitution of the sublayer does not strongly affect catalytic performance. To validate this hypothesis, we performed density functional theory (DFT) calculations of the electronic structure. Projected density of states analysis revealed that the density near the Fermi level is dominated by Fe d-orbitals, confirming that the electrochemical behavior is largely governed by Fe rather than the choice of Ge or Ga (Figure 2d) (more detailed DOS calculations are provided in the Supplementary Materials).

**Figure 3:**
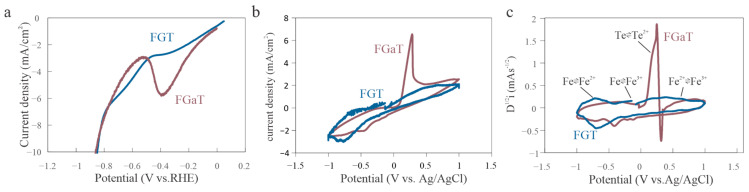
Electrochemical comparison of homologs. (**a**) LSV curve revealing a distinct peak of FGaT at approximately −0.3 V vs. RHE. (**b**) Capacitive-background-corrected cyclic voltammetry (CV) curves showing a stronger anodic current response in FGaT. (**c**) Semi-derivative CV analysis identifying key redox transitions in FGT (at −0.6 V, −0.2 V, and 0.6 V) and a Te-related oxidation peak in FGaT at 0.3 V.

Yet, closer investigation of the LSV data shows that this interpretation is incomplete. An unexpected peak in the FGaT curve at approximately −0.4 V vs. RHE occurs (Figure 3a). Although this feature has been previously seen in studies of exfoliated FGT, it has not been investigated [25]. To further probe this anomaly, we conducted cyclic voltammetry (CV) on both materials in the same electrolyte. To ensure accurate comparison, the CV curves were normalized by the capacitive background charge, accounting for differences in crystal surface area. The resulting curves were largely similar, but a pronounced deviation was observed in the anodic branch of FGaT, suggesting a fundamental difference in oxidative behavior (Figure 3b).

To quantify this discrepancy, we performed a semi-derivative analysis of the CV response (Figure 3c). For FGT, three distinct peaks appeared at −0.6 V, −0.2 V, and 0.6 V vs. Ag/AgCl, corresponding to sequential oxidation states of Fe and underscoring its electrochemical significance [35,36]. In contrast, FGaT’s anodic current was dominated by a peak at 0.3 V, which was attributed to the oxidation of Te [37]. These results indicate that while Ga does not directly participate in the electrochemical reaction, it influences the stability of surface Te atoms.

**Figure 4:**
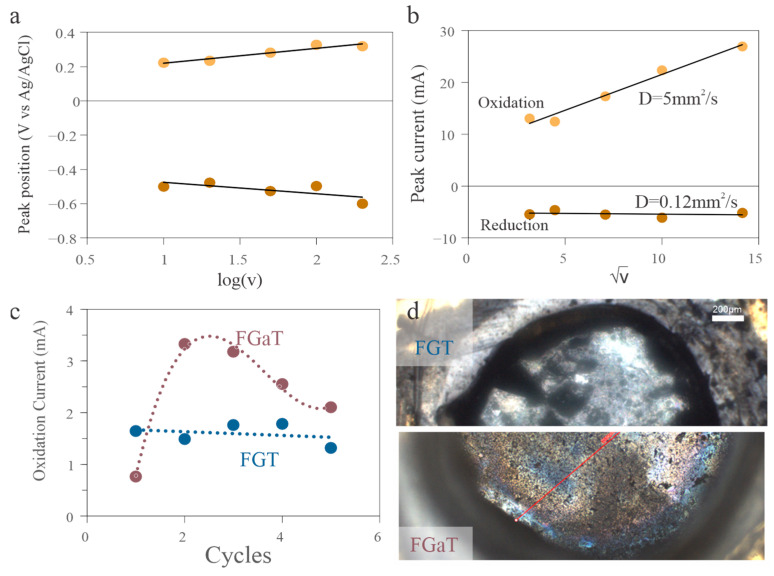
Electrochemical degradation behavior. (**a**) CVs at different scan rates, showing increasing peak separation with scan rate, indicating kinetic limitations. (**b**) Scan rate–dependent analysis of peak current, revealing diffusion-limited and irreversible oxidation behavior. (**c**) Cycling data comparing performance degradation in FGT and FGaT. (**d**) Horizontally split arrangement of two optical microscopy images post-cycling, showing retention of flat surface in the FGT case (**top**) and visible degradation in FGaT (**bottom**), consistent with surface corrosion.

To explore this hypothesis, we conducted scan-rate-dependent CV experiments that help assess the reversibility of Te oxidation. As the scan rate increased, the separation between the anodic and cathodic peaks widened for both materials, indicating kinetic limitations (Figure 4a). We quantify this effect by plotting the peak current versus scan rate, which reveals a distinct difference in behavior between the oxidation and reduction branches (Figure 4b) [38]. Application of the Randles–Sevcik equation shows that the diffusion coefficient for oxidation was approximately 50 times higher than that for reduction, confirming the irreversibility of the Te oxidation process.

To understand why this instability was more pronounced in FGaT, we performed DFT calculations comparing the formation energy of Te vacancies in both materials. Using experimentally derived lattice parameters, we computed the total energies of the pristine FGT and FGaT configurations (E_pristine_) in a single unit cell. We then subtracted the energies of the structure when the Te atom was removed (E_deficient_) and when an isolated Te atom (E_Te_) was retained in the unit cell, written as follows:

(1) $$\Delta E=E_{pristine}-E_{deficient}-E_{Te}$$

Our calculations revealed that the energy of Te removal in FGaT was approximately 4.16 eV, whereas FGT exhibited a vacancy formation energy of 4.36 eV. This energy difference of 200 meV indicates that Te is more weakly bound in the Ga-containing structure.

To experimentally validate these findings, we subjected both materials to prolonged electrochemical cycling. FGT demonstrated significantly better stability, with lower performance degradation compared to FGaT (Figure 4c). Post-mortem optical inspection confirmed that this degradation in FGaT was accompanied by visible physical damage, consistent with Te loss and structural breakdown during oxidation (Figure 4d).

## 4. Conclusions

In conclusion, we have provided a comparative analysis of two novel layered materials, FGT and FGaT. The structural and morphological similarity was established by diffraction and microscopic analysis. The similarity in surface structure results in quasi-identical hydrogen evolution reactions as explained by the dominance of iron in the electrochemical response. However, the stability of FGaT was found to be less than FGT and theoretically explained by a lowered Te stability. Our results emphasize the value of structural homologs in magnetic 2D materials and the importance of considering stability for envisioned magneto-electrochemistry.

## Data Availability

The data that support the findings of this study are available from the corresponding author upon reasonable request.

## Supplementary Materials

The supporting information can be downloaded at https://www.mdpi.com/article/10.3390/nano15151210/s1. Figure S1: SEM image of surface of (a) Fe_3_GeTe_2_ (b) Fe_3_GaTe_2_, Figure S2: Optical Microscope image of (a) Fe_3_GeTe_2_ (b) Fe_3_GaTe_2_ with silicone mask covering, Figure S3: Device Schematic, Figure S4: CV’s of FGaT at different scan rates, Table S1: Peak Positions of FGaT CV, Figure S5: CV’s of FGT at different scan rates, Table S2: Peak Positions of FGT CV, Table S3: Bond Lengths by Type and Strain Condition, Figure S6: Spin-resolved density of states calculation for both materials under different amounts of strain was calculated using Quantum ATK’s LCAO-based density functional theory (DFT), employing the generalized gradient approximation (GGA) with norm-conserving pseudopotentials.
