# Supplementary material for: Uncovering Stability Origins in Layered Ferromagnetic Electrocatalysts Through Homolog Comparison
Source: Nanomaterials (Basel). 2025 Aug 7;15(15):1210. doi: 10.3390/nano15151210 (PMC12348357; doi:10.3390/nano15151210)
Supplement: Supplementary file 1 [file nanomaterials-15-01210-s001.zip › nanomaterials-3749920-supplementary.pdf]

Supplementary Materials

Uncovering Stability Origins in Layered Ferromagnetic Electrocatalysts through Homolog Comparison

Om Prakash Gujela [1,2], Sivasakthi Kuppusamy [3], Yu-Xiang Chen [4,5,6], Chang-Chi Kao [7], Jian-Jhang Lee [4], Bhartendu Papnai [8,9], Ya-Ping Hsieh [4], Raman Sankar [3] and Mario Hofmann [7,*]

[1] Graduate Institute of Applied Physics, National Taiwan University, Taipei 10617, Taiwan; opgujela@gmail.com
[2] Department of Electrical and Electronics Engineering, Bakhtiyarpur College of Engineering, Bihar Engineering University, Patna 800001, India
[3] Institute of Physics, Academia Sinica, Taipei 11529, Taiwan; sivasaran413@gmail.com (S.K.); sankarndf@gmail.com (R.S.)
[4] Institute of Atomic and Molecular Sciences, Academia Sinica, Taipei 10617, Taiwan; berry45670@gmail.com (Y.-X.C.); jonaslee20@gmail.com (J.-J.L.); yphsieh@gate.sinica.edu.tw (Y.-P.H.)
[5] International Graduate Program of Molecular Science and Technology, National Taiwan University, Taipei 10617, Taiwan
[6] Molecular Science and Technology Program, Taiwan International Graduate Program, Academia Sinica, Taipei 10617, Taiwan
[7] Department of Physics, National Taiwan University, Taipei 106319, Taiwan; r13222052@g.ntu.edu.tw
[8] Department of Engineering and System Science, National Tsing Hua University, Hsinchu 300044, Taiwan; bharatpapnai@gmail.com
[9] Nanoscience and Technology Program, Taiwan International Graduate Program, Academia Sinica, Taipei 10617, Taiwan
* Correspondence: mario@phys.ntu.edu.tw

Description of Growth Setup

In our experimental setup for chemical vapor transport (CVT) growth, we used two concentric tubes of different sizes to create a controlled and effective environment for crystal synthesis. A smaller quartz tube (both in diameter and length) was placed inside a larger outer quartz tube. The starting materials (approximately three grams) were loaded into the smaller inner tube, which was left unsealed. The larger outer tube was then evacuated using a vacuum pump and subsequently flame-sealed, ensuring that all reactions occurred in a vacuum atmosphere. This design served two key purposes. First, the inner tube held the reactants without needing to be sealed, while the outer tube maintained the overall vacuum necessary for clean vapor-phase reactions. Second, the outer tube has a sufficient length to establish a temperature gradient—with a hot zone where sublimation occurs and a cool zone where deposition and crystal growth take place. Iodine, used as the transport agent, facilitated the movement of material in vapor form from the hot to the cool zone, where it crystallized.

In our chemical vapor transport (CVT) experiments, no inert gas flow was used during the growth process. Instead, a vacuum environment was maintained inside the sealed quartz tube. The tube was evacuated using a vacuum pump and flame-sealed to ensure that all reactions occurred under low-pressure, oxygen-free conditions, which are critical for effective transport and high-quality crystal growth.

Scanning Electron Microscope

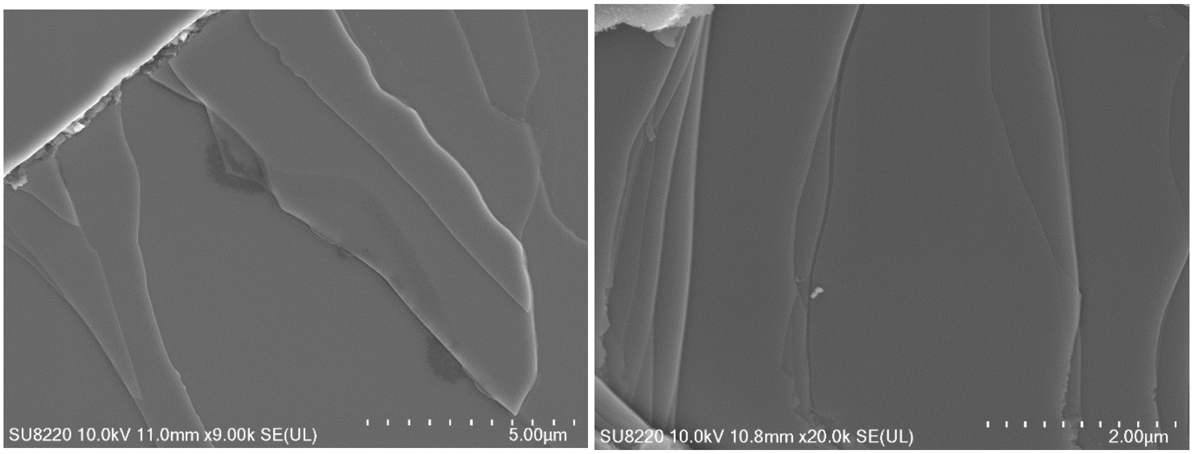

Figure S1. SEM image of the surfaces of (a) Fe_3GeTe_2 and (b) Fe_3GaTe_2.

Optical Microscope Image:

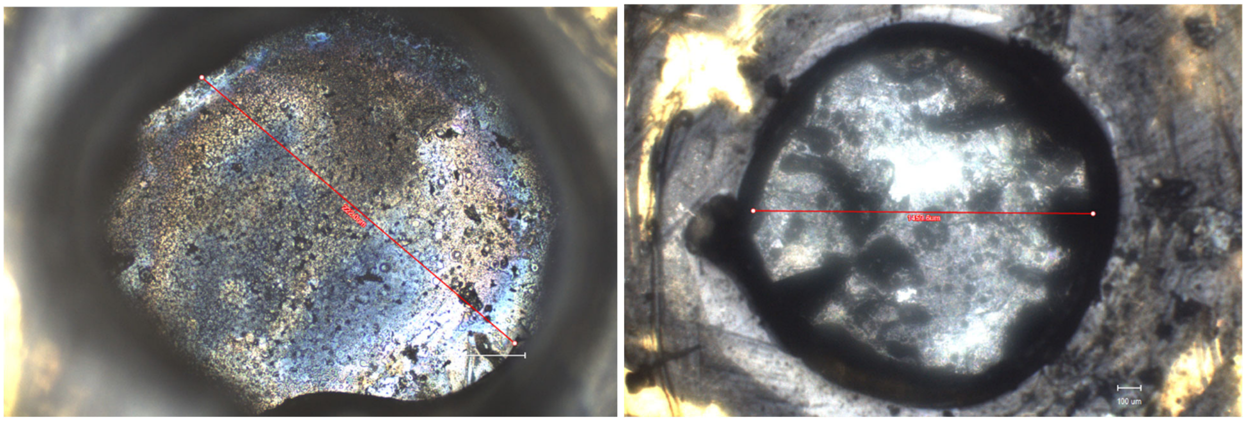

Figure S2. Optical microscope images of (a) Fe_3GeTe_2 and (b) Fe_3GaTe_2 with silicone mask covering.

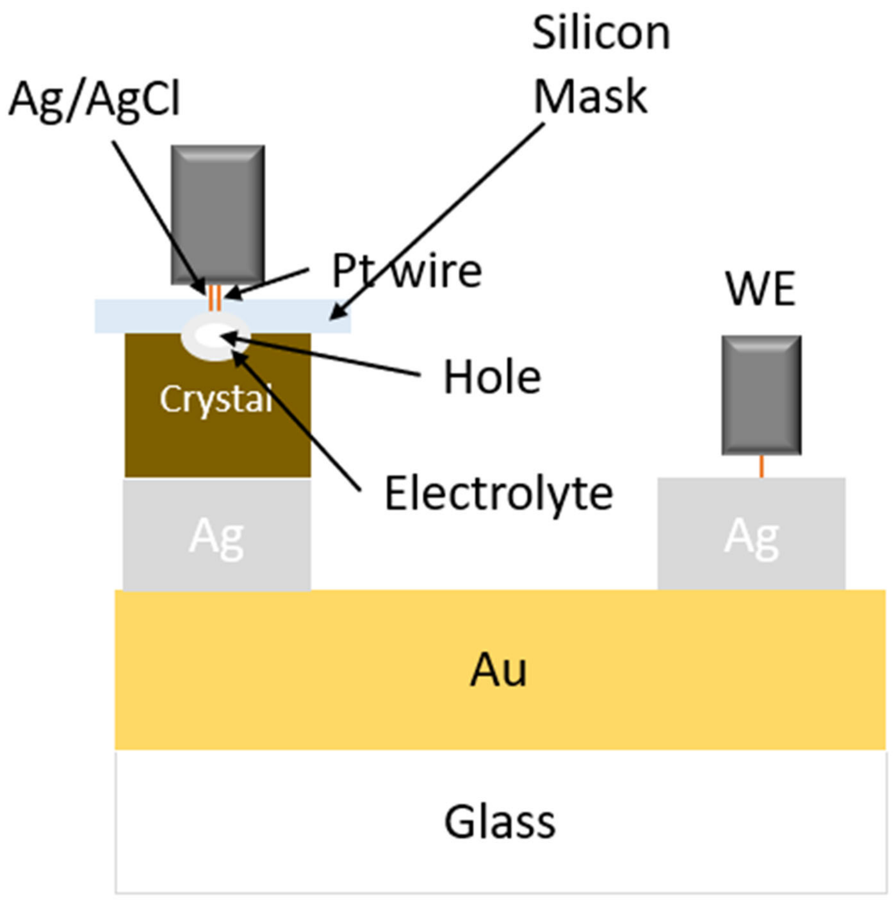

Figure S3. Device schematic.

Cyclic Voltammetry Curve:

1. Fe3GaTe2 (0.5 M of H2SO4 electrolyte)

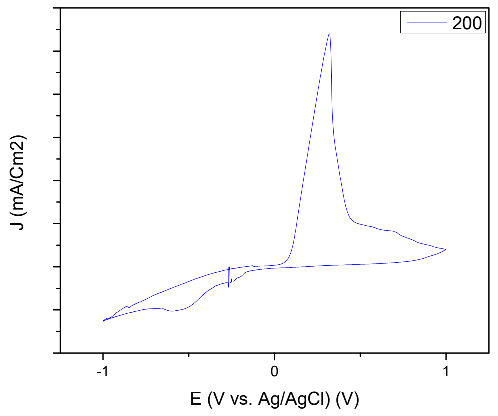

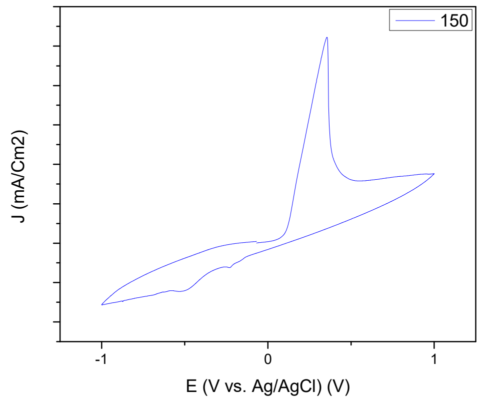

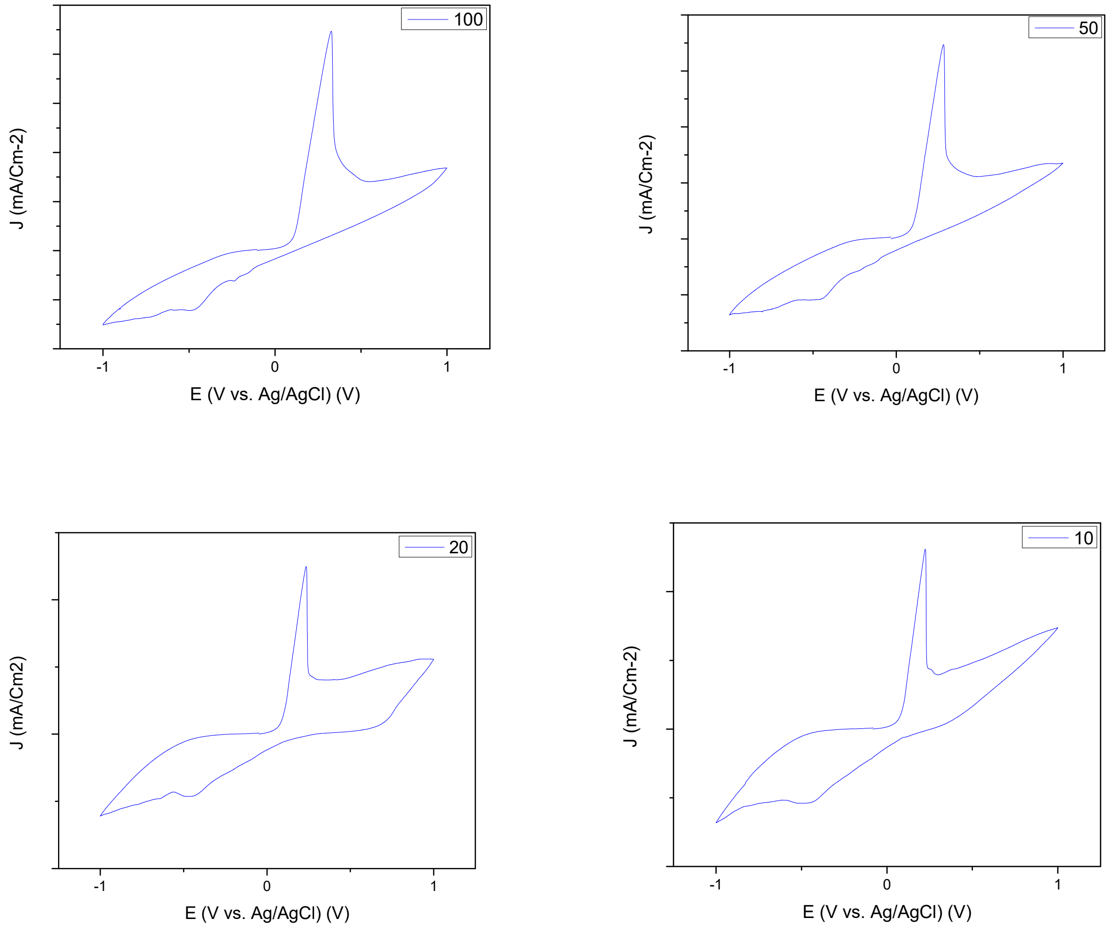

Figure S4. CV's of FGaT at different scan rates.

Table S1. Peak positions of FGaT CV.

**Scan Rate**	**Peak Position Oxidation (Upper Curve)**		**Peak Position Reduction (Lower Curve)**	
	X (Voltage)	Y (mACm-2)	X (Voltage)	Y(mACm-2)
**10**	0.22384	13095.03139	-0.49997	-5402.76582
**20**	0.23464	12477.51915	-0.476	-4637.4664
**50**	0.28274	17351.85147	-0.52546	-5470.51633
**100**	0.32735	22348.54832	-0.49693	-6100.09618
**150**	0.35492	26129.55127	-0.53242	-6120.31538
**200**	0.3195006	26992.2418	-0.59905	-5146.76035

2. Fe3GeTe2 (0.5 M of H2SO4 electrolyte)

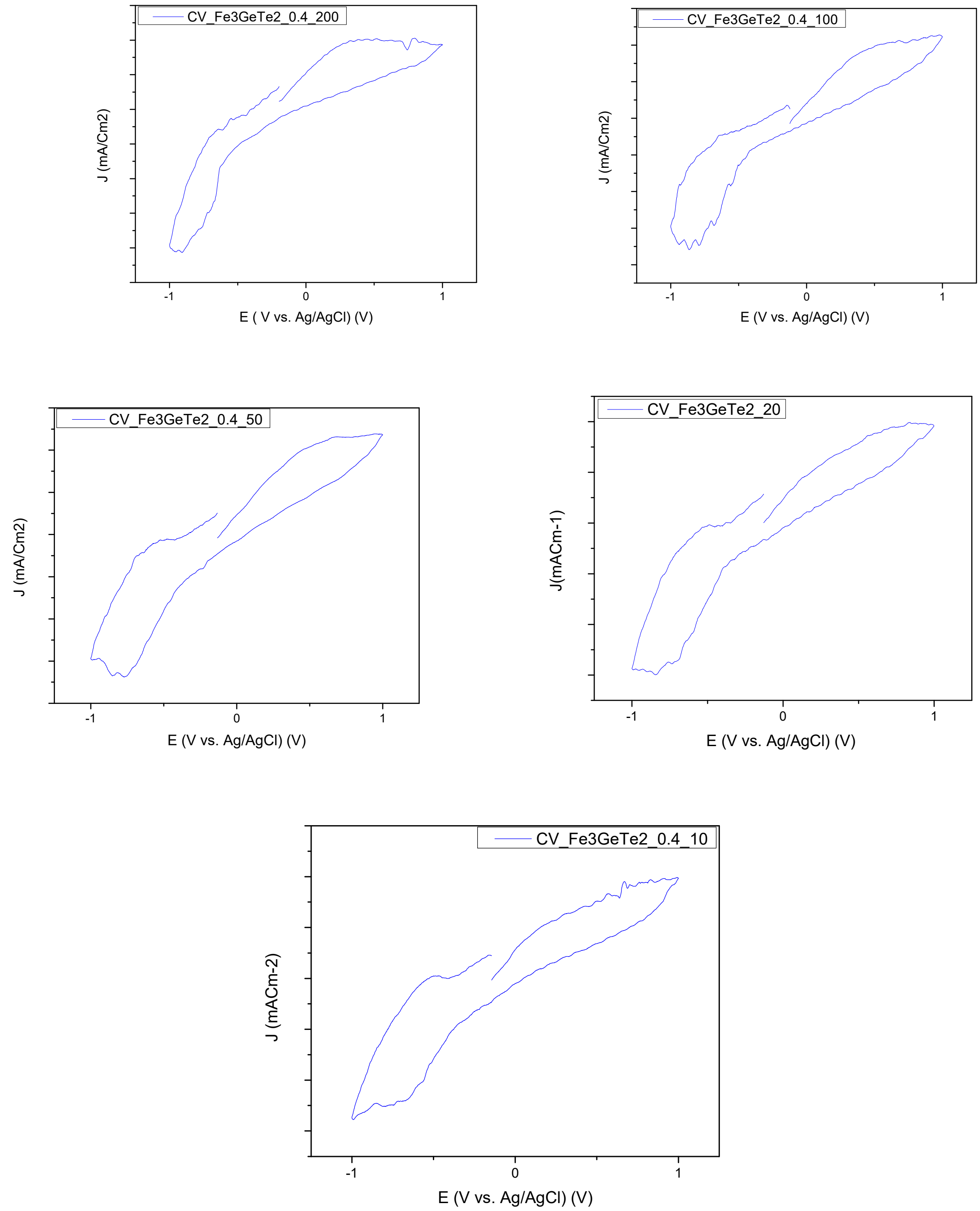

Figure S5. CV’s of FGT at different scan rates.

Table S2. Peak positions of FGT CV.

Scan Rate	Peak Position Oxidation (Upper Curve)		Peak Position Reduction (Lower Curve)	
	X (Voltage)	Y (mACm-2)	X (Voltage)	Y(mACm-2)
**10**	-0.48507	4.911E-5	-0.80728	-0.00252
**20**	-0.49369	-3.9969E-5	-0.84683	-0.00299
**50**	-0.52458	-1.25423E-4	-0.77027	-0.00338
**100**	-0.6415	-4.65478E-4	-0.86113	-0.00359
**200**	-0.63899	-5.67081E-4	-0.91178	-0.00413

Table S3. Bond lengths by type and strain condition.

Strain-dependent crystal structures were simulated in QuantumATK by uniformly expanding the unit cells of relaxed FGT and FGaT structures, followed by structural relaxation with a force tolerance of 0.05 eV/Å.

FGT (Iron–Germanium–Tellurium)

Bond Type	-5% Compressive (Å)	+5% Tensile (Å)	Absolute Change (Å)	Relative Change (%)
**Te-Fe**	2.54	2.81	+0.27	+10.6%
**Fe-Ge (in-plane)**	2.61	2.88	+0.27	+10.3%
**Fe-Ge (interlayer)**	2.67	2.95	+0.28	+10.5%

FGaT (Iron–Gallium–Tellurium)

Bond Type	-5% Compressive (Å)	+5% Tensile (Å)	Absolute Change (Å)	Relative Change (%)
**Te-Fe**	2.54	2.81	+0.27	+10.6%
**Fe-Ga (in-plane)**	2.61	2.88	+0.27	+10.3%
**Fe-Ga (interlayer)**	2.67	2.95	+0.28	+10.5%

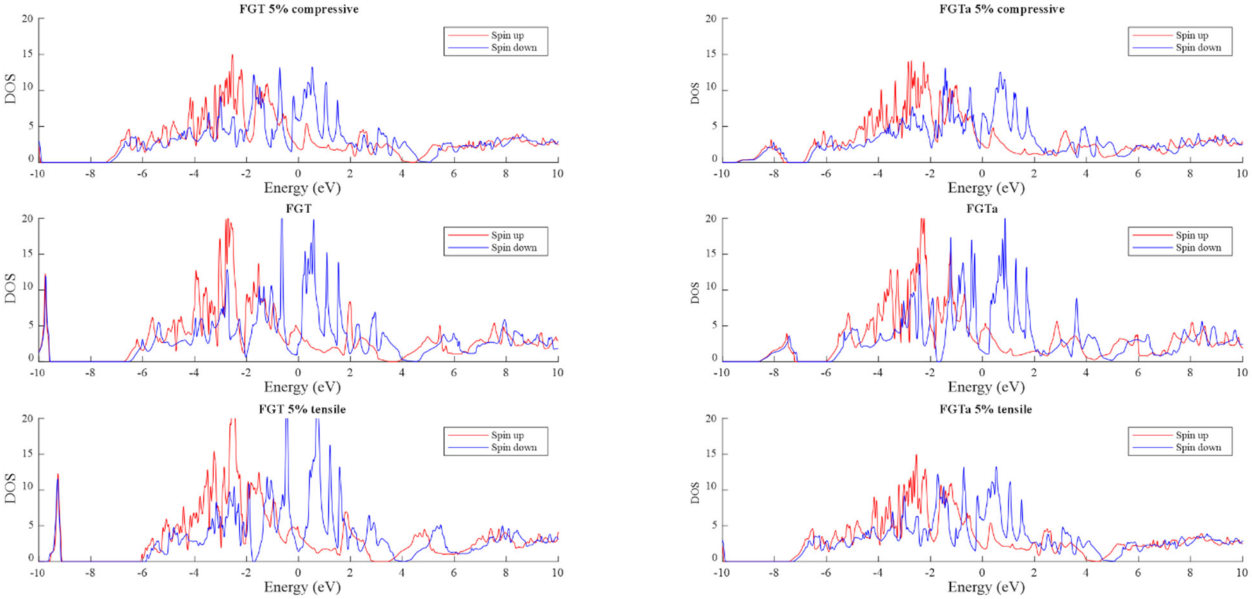

Figure S6. Spin-resolved density of states calculation for both materials under different amounts of strain was calculated using QuantumATK's LCAO-based density functional theory (DFT), employing the generalized gradient approximation (GGA) with norm-conserving pseudopotentials.
